# Himalayan Marmot (*Marmota himalayana*) Redistribution to High Latitudes under Climate Change

**DOI:** 10.3390/ani13172736

**Published:** 2023-08-28

**Authors:** Zhicheng Wang, Yukun Kang, Yan Wang, Yuchen Tan, Baohui Yao, Kang An, Junhu Su

**Affiliations:** 1 College of Grassland Science, Gansu Agricultural University, Lanzhou 730070, China; zhicheng_wang0910@163.com (Z.W.); kang_an1997@163.com (K.A.); 2 Key Laboratory of Grassland Ecosystem (Ministry of Education), Gansu Agricultural University, Lanzhou 730070, China; 3 Gansu Agricultural University-Massey University Research Centre for Grassland Biodiversity, Gansu Agricultural University, Lanzhou 730070, China

**Keywords:** climate change, Qinghai–Tibet Plateau, Himalayan marmot, plague, maximum entropy model, centroid migration

## Abstract

**Simple Summary:**

The Himalayan marmot is a host animal for plague-causing pathogens and is endemic to the Qinghai–Tibet Plateau (QTP). With human activities and global warming, the Himalayan marmot population has increased rapidly, causing damage to grassland ecosystems and increasing the risk of plague infection in humans. The current and future potential distribution of the Himalayan marmot has significant implications for ecosystem management, biodiversity conservation, and public health safety on the QTP. The maximum entropy model was employed to analyze the Himalayan marmot potential distribution in the near and later future (2050s and 2070s) in this study. Under future climate scenarios, the uninterrupted rise in precipitation and temperature over the QTP predicted that the suitable area for the Himalayan marmot will increase, with the centroid shifting to higher latitudes. Our results indicate that climatic factors are of great significance for the distribution of the Himalayan marmot and provide a reference for ecosystem management and the monitoring of plague epidemics in the QTP.

**Abstract:**

Climate warming and human activities impact the expansion and contraction of species distribution. The Himalayan marmot (*Marmota himalayana*) is a unique mammal and an ecosystem engineer in the Qinghai–Tibet Plateau (QTP). This pest aggravates grassland degradation and is a carrier and transmitter of plagues. Therefore, exploring the future distribution of Himalayan marmots based on climate change and human activities is crucial for ecosystem management, biodiversity conservation, and public health safety. Here, a maximum entropy model was explored to forecast changes in the distribution and centroid migration of the Himalayan marmot in the 2050s and 2070s. The results implied that the human footprint index (72.80%) and altitude (16.40%) were the crucial environmental factors affecting the potential distribution of Himalayan marmots, with moderately covered grassland being the preferred habitat of the Himalayan marmot. Over the next 30–50 years, the area of suitable habitat for the Himalayan marmot will increase slightly and the distribution center will shift towards higher latitudes in the northeastern part of the plateau. These results demonstrate the influence of climate change on Himalayan marmots and provide a theoretical reference for ecological management and plague monitoring.

## 1. Introduction

It is expected that climate change will have a significant impact on biodiversity [1]. In recent years, global warming has become a major challenge for human and biological communities by affecting the living environment, habitat, distribution range, and quantity of species; interspecific relationships; population size; and ecosystem structure and function [2,3,4,5,6], and the rate of species extinction is dramatically accelerating [7,8,9], which would significantly affect biodiversity. Therefore, accurately predicting the effect of climate change on species survival is crucial for policymakers to assess threats to biodiversity and respond proactively.

Some studies have found that continued global warming may force certain species to migrate to higher altitudes or latitude [10,11,12]. Conversely, if species cannot migrate because of geographical barriers, they may experience population differentiation and even population extinction [13,14,15]. Species that are particularly sensitive to climate change, such as rodents, possess specific characteristics, such as shorter lifespans and higher fecundity, and are considered indicators of climate change [16,17]. They rely on vegetation and seeds as their food and are preyed on by various predators; therefore, they play essential roles as intermediate nutrients and indicators in the ecosystem to sustain the balance of the ecosystem and protect biodiversity [18].

Moreover, human activity, such as increasing the area of arable and pasture land, has altered land-use patterns and is also a main driver of the potential distribution of species, which has resulted in a decline in species population [19]. Since the 1970s, the influence of human activities on all aspects of interactions between species and their environments has increased dramatically on a global scale and have macro-level impacts on the environment, such as species distribution [20], population dynamics, and viability [21]. Human activities also limit species ability to respond to climate change by affecting their migration and diffusion, which results in the shrinkage or loss of habitat for various species [2,22,23]. 

Plague is a deadly human disease caused by the gram-negative bacterium *Yersinia pestis* and can present as septicemic, pneumonic, and bubonic plague and bubonic plague types [24,25,26]. It is mainly prevalent in the Qinghai–Tibet Plateau (QTP), Inner Mongolia Plateau, and Songliao Plain [27], among which the QTP is the largest and has the highest-altitude epidemic focus. In recent years, plague infection in humans and grazing livestock have frequently occurred in the QTP region [28,29], seriously endangering the local social and economic development and production activities. However, the QTP is vast in area, and the statistics of epidemic focal points based on administrative divisions do not reflect the true spatial distribution of plagues in the QTP region, nor can they provide effective guidance for the routine monitoring of epidemic focal points [30]. Some studies have used geographic information systems and niche modeling to determine the spatial distribution of host animals in China [29,30,31]. Compared with traditional surveys, this method is not only efficient and time-saving but also has the advantages of scientific objectivity [32].

The Himalayan marmot (*Marmota himalayana*) is a member of the Rodentia order and Sciuridae families, and of the *Marmota* genus [33]. It is an endemic species to the QTP and is an important source of human plague in this area, explaining 73.17% of human plague infections [34,35]. Moreover, the Himalayan marmot causes other damage by digging holes that destroy grass roots and turf. The plants surrounding the holes get covered by soil, which increases the risk of grassland degradation [36]. However, as part of the ecosystem, the Himalayan marmot plays a crucial role in maintaining the stability of alpine meadow. They serve as a significant food source for some large raptors and carnivores, and their digging activities facilitate the organic matter cycle [37]. Therefore, understanding the spatial distribution and future distribution patterns of the Himalayan marmot is of great significance for ecosystem stability, biodiversity conservation, and public health security in the QTP.

In the present study, the maximum entropy (MaxEnt) model was employed to evaluate the spatial distribution pattern of the Himalayan marmot across the entire QTP, reveal the potential effect of climate change on the Himalayan marmot, and compare the changes in their suitable areas under different climate scenarios for projected socio-economic global changes. Our objectives were to determine (1) the environmental parameters and habitat preferences that influence the distribution of Himalayan marmots, (2) the distribution patterns of Himalayan marmots under different shared socio-economic pathways, and (3) whether the Himalayan marmots are likely to shift to higher altitudes or latitudes in the future. The purpose of the present study was to provide a reference for species management, plague prevention, and control of the Himalayan marmot in the QTP.

## 2. Materials and Methods

### 2.1. Study Area

**Figure 1:**
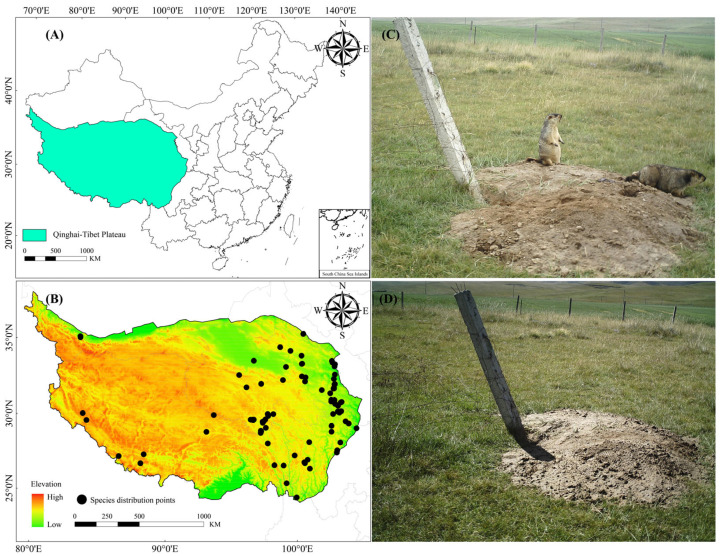
Study area and species distribution points in the Qinghai–Tibet Plateau. (**A**) The Qinghai–Tibet Plateau; (**B**) the Himalayan marmot distribution site; (**C**) Himalayan marmots (captured by infrared camera in Tianzhu Tibetan Autonomous County, China, on 3 September 2021); and (**D**) Grassland landscape inhabited by Himalayan marmots (captured by infrared camera in Tianzhu Tibetan Autonomous County, China, on 3 September 2021).

The QTP is located in southwest China (73.43°~104.67° E, 25.98°~39.82° N) and includes Tibet and Qinghai, Gansu, Sichuan, Yunnan, and Xinjiang provinces (Figure 1A); it has a total area of about 2.5 × 10^6^ km^2^ and an average altitude of about 4500 m [38], making it the highest plateau in the world. The QTP has very complex climatic conditions due to the special geographical location and environment of the QTP [39]. The climate of the plateau is cold and dry in the northwest and warm and wet in the southeast, with precipitation mainly concentrated in the south of the plateau [40]. Because of its spatial differences in hydrothermal conditions and large elevation range, the QTP forms a variety of ecosystem types, including forests, grasslands, and deserts that provide excellent habitats for a wild variety of numerous wildlife species [41,42,43]. The QTP also has many numerous rare species and endemic species, such as the Tibetan antelope (*Pantholops hodgsonii*), Tibetan fox (*Vulpes ferrilata*), black-necked crane (*Grus nigricollis*), snow leopard (Panthera uncia), Himalayan marmot, plateau pika (*Ochotona curzoniae*), and plateau zokor (*Eospalax baileyi*). Therefore, the QTP is known as a “natural habitat for rare animals and a gene pool of plateau life” [44].

### 2.2. Occurrence Data Collection

According to the following sources, the distribution sites of Himalayan marmots were obtained for this study: (1) China Zoology Database (http://www.zoology.csdb.cn, accessed on 24 June 2023); (2) Global Biodiversity Information Facility (GBIF, https://www.gbif.org, accessed on 24 June 2023) [45]; (3) China National Knowledge Infrastructure database (CNKI, https://www.cnki.net, accessed on 25 June 2023) [45,46]; and (4) GPS sites records during our field surveys from 2001 to 2021. Before the analysis, we manually omitted points with unknown records and imported ENMTools software v1.4.4 to remove duplicates and adjacent occurrence records, and, finally [47], we separately collected 93 valid record distribution points in the QTP to plot their distribution patterns (Figure 1B; Table S1).

### 2.3. Environmental Variables

Current bioclimatic variables were obtained from WorldClim (http://www.worldclim.org, accessed on 26 May 2023), which supplies mean climate data from global and regional weather stations from 1970 to 2000, and we also gained the elevation data from this website [48]. For the future climate data, we selected the Beijing Climate Center-Climate System Model-Medium Resolution (BCC-CSM2-MR), which is one of the most commonly used models in China [41,49,50,51]. In our study, three climate scenarios (shared socio-economic pathways, SSPs) (Low: SSP1-2.6; Medium: SSP2-4.5; High: SSP5-8.5) were used to forecast the Himalayan marmot potential distribution between 2041 and 2060 (2050s) and 2061 and 2080 (2070s) [52]. We also downloaded the human footprint index (HFI) from the archives (http://sedac.ciesin.columbia.edu/data, accessed on 30 May 2023). This dataset was obtained by combining the impacts of human activities such as land use, population density, railways, roads, power infrastructure, arable land, and grazing [53]. After the coordinate system and spatial resolution (2.5 arc-minutes) of all environmental variables were unified, the mask was extracted to the QTP region, and all variables were output as “ASCII” format in ArcGIS software (ESRI, Redlands, CA, USA, https://desktop.arcgis.com, accessed on 15 January 2022, v10.8) for subsequent analysis. To avoid overfitting of the model because of multicollinearity among environmental variables, all variables were analyzed using correlation analysis in the ENMTools software, and then variables with a correlation coefficient |*r*| < 0.8 were selected for subsequent modeling [47] (Table S2). Finally, 10 environmental variables were obtained for ecological niche modeling in this study (Table S3).

### 2.4. Suitable Areas Distribution Modeling

We used MaxEnt v3.3.4 to develop the species distribution models (SDMs) [54]. The MaxEnt model can outperform other models [55,56], and its low sensitivity advantages of environmental variables collinearity allow it to remain stable, even when the sample size is small, and fit complex variable relationships [57,58]. The feature combination (FC) parameters and regularization multiplier (RM) were optimized by the “ENMeval” package in our model [59]. The model was adjusted by setting the six FCs (L, LQ, H, LQH, LQHP, and LQHPT; L = linear, Q = quadratic, H = hinge, P = product, and T = threshold) and RMs (0.5, 1.0, 1.5, 2.0, 2.5, 3.0, 3.5 and 4.0) [59]. Using different parameter conditions, the ENMeval data package tested the Akaike information criterion correction (AICc) values that were corrected by Maxent (AICc) to determine the complexity [60]. As a standard for measuring the validity of statistical model fitting, the AIC generally prioritizes parameters with small AIC values. Finally, we used the receiver operating curve (ROC) method, area under the curve (AUC) values, and the continuous Boyce index (CBI) to evaluate the model performance [51,61].

When predicting the model, 75% of the distribution points and the remaining points were selected randomly as the training dataset to evaluate the prediction effect of the model [62], and the optimized RM and FCs (RM = 3, FC = H) were modified (Table S4). The average was selected as the final simulation result [63]. We used the natural breaks (Jenks) method to reclassify the average suitable area of species into four suitable grades [64]: high suitable area (HSA), medium suitable area (MSA), low suitable area (LSA), and unsuitable area (USA). The area of all levels was calculated by a raster calculator in ArcMap.

### 2.5. Superposition Analysis of High Suitability Area for Himalayan Marmots and Land-Use Types

**Figure 2:**
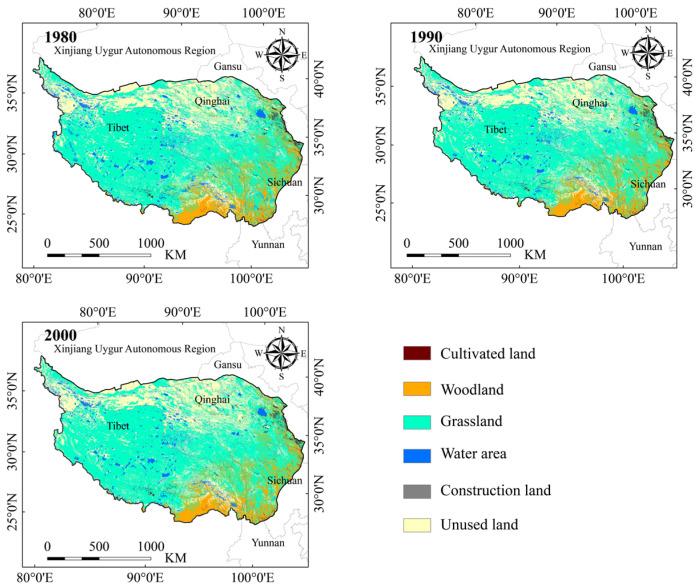
Land-use types of the Qinghai–Tibet Plateau in three time periods.

To understand the choice of land-use type preferences in the Himalayan marmot, we analyzed the HSA and compared the land-use types in the three different time periods, which were obtained from the Resource and Environment Science and Data Center of the Chinese Academy of Sciences (https://www.resdc.cn, accessed on 26 June 2023) [65]. In order to match the current climate scenario data in time as much as possible (the time of the contemporary climate data raster layer was 1970–2000), we used the land-use types in 1980,1990, and 2000 for analysis (Figure 2). The data of land use from these three periods was employed to calculate the area of different land-use types in the Himalayan marmot HSA.

### 2.6. Migration Trend Analysis of Himalayan Marmot

To analyze the vector distance and direction of the centroid of the Himalayan marmots in future climates, the SDM Toolbox in the ArcMap software v10.8 was used to simulate the centroid from the current distribution to the future distribution in the QTP [66]. Here, species distributions are represented by centroids, and changes in species distributions over time are displayed by vectors [67]. The distribution changes of the Himalayan marmot on the QTP were monitored by estimating changes in the location of the future distribution centroid for each of the SSPs assessed. We also calculated the distance between the centroids as previously described [68].

## 3. Results

### 3.1. Analyses of Environmental Variables

**Figure 3:**
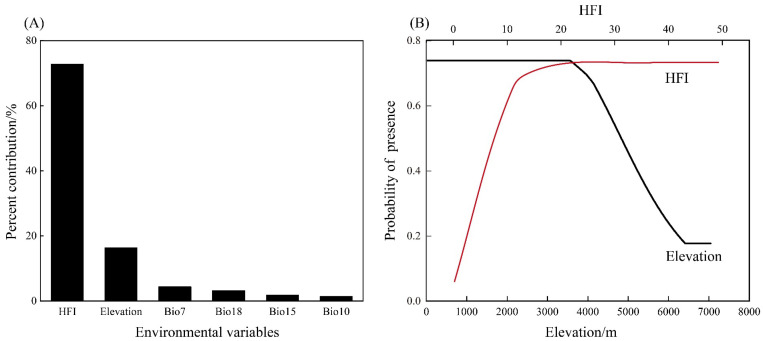
Influence of environmental factors on the distribution of Himalayan marmot. (**A**) Contribution rate of environmental variables. (**B**) Response curves of main environmental variables.

The MaxEnt model performed well, with AUC and CBI values of 0.861 and 0.944, respectively (Table S4). The results indicated that the relative contribution rate of environmental variables to the Himalayan marmot was different from that of our model. The HFI accounts for 72.80% of the effects on Himalayan marmot distribution and an elevation of 16. 40%, whereas the effects of the other environmental factors on the potential distribution of the Himalayan marmot were low (Figure 3A). We plotted the response curves for the main environmental factors of the model and found that the presence probability of the Himalayan marmot increased with HFI, remained constant when the altitude was less than 3542.39 m, and decreased when the altitude was greater than this value. As the probability of the presence of the Himalayan marmot increased, the degree of HFI gradually increased, whereas HFI = 20.56 correlated with a constant probability of Himalayan marmot existence (Figure 3B).

### 3.2. Suitable Areas for the Himalayan Marmot

**Figure 4:**
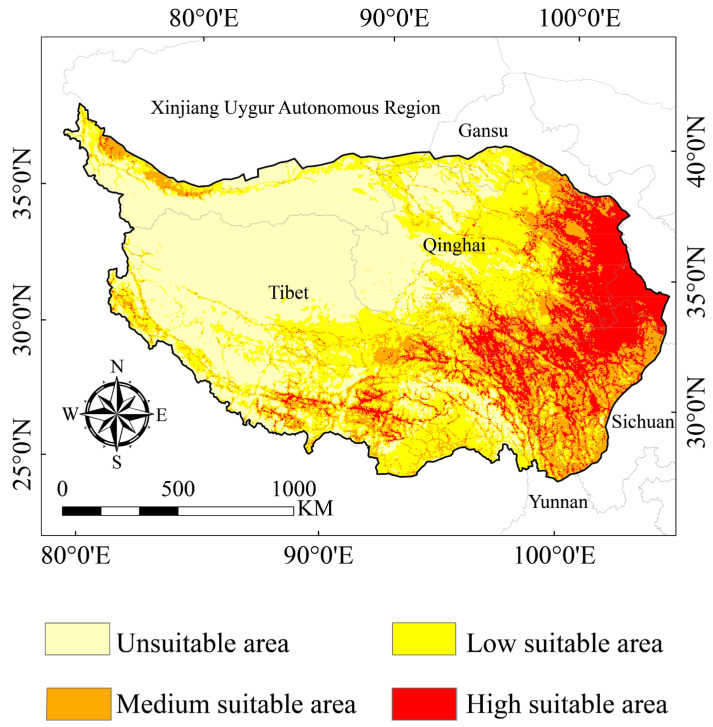
Current potential distribution of the Himalayan marmot.

In our model, the HSA of the Himalayan marmot was mainly distributed in the eastern, southern, and northwestern regions of the QTP, including most parts of the Tibet Autonomous Region and Qinghai, Gansu, and Sichuan provinces, as well as the cities of the Xinjiang Uygur Autonomous Region. The MSA of the Himalayan marmot was mainly distributed in the northeastern and central parts of Qinghai Province and the southern and central parts of Tibet (Figure 4). 

### 3.3. Superposition Analysis of Land-Use Types and High Suitable Area

By stacking the land-use types for the three time periods (Figure 2) and the HAS (Figure 4), we found that there was little variance in the choice preference of land-use types of Himalayan marmots between the three periods studied. However, grassland was the preferred habitat of Himalayan marmots on the QTP, with a mean value of 2.12 × 10^5^ km^2^ for their preferred habitat selection area. The area of woodlands in the HSA during the three time periods were 7.91 × 10^4^ km^2^, 7.92 × 10^4^ km^2^, and 7.90 × 10^4^ km^2^, respectively. The Himalayan marmot also had a certain preference for cultivated land; the area of cultivated land categorized as HSA in the three tested time periods were 1.65 × 10^4^ km^2^, 1.60 × 10^4^ km^2^, and 1.61 × 10^4^ km^2^, respectively. By calculating the secondary classification area of grassland types, we found that the Himalayan marmot preferred the grassland types with medium coverage, which were 9.68 × 10^4^ km^2^, 1.03 × 10^5^ km^2^, and 9.67 × 10^4^ km^2^ respectively, in the time periods used in the analysis model (Figure 2 and Figure 4, Tables S5 and S6).

### 3.4. Suitable Habitat Area and Centroid Changes under Future Climate Scenarios

**Figure 5:**
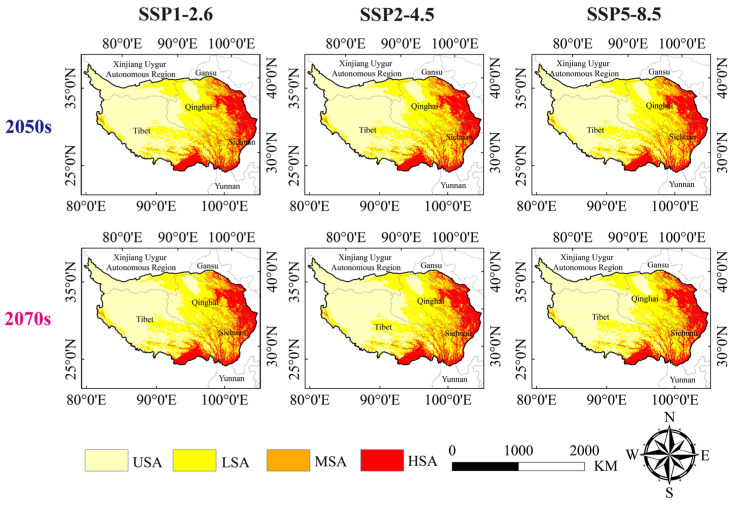
The potential distribution of Himalayan marmots in the Qinghai–Tibet Plateau under different climate scenarios.

When compared with the suitable area level of marmots, the future average areas of the HSA, LSA, and USA of the Himalayan marmots in different shared socio-economic paths increased (2050s: HSA: 7.89%; LSA: 4.19%; USA: 7.10%; 2070s: HAS: 7.73%; LSA: 5.69%; USA: 6.25%). However, the MSA area decreased, with an average reduction of 29.54% and 29.74% in the 2050s and 2070s, respectively. We also found that the areas of suitable habitat that were predicted to increase in the future were mainly in the central and northern parts of the Qinghai Province and the southeastern regions of the Tibet Autonomous Region. On the other hand, the areas of suitable habitat that were predicted to decrease were mainly in the northwestern and central regions of the QTP (Figure 3B and Figure 5, Table S7). Through the analysis of the future climate scenarios for Himalayan marmot suitable habitats, we found that the centroid position of the 2050s and 2070s in different shared socio-economic paths of mass had exactly the same latitude and longitude (2050s: longitude: 99.02051, 33.01076; 2070s: longitude: 99.030034, latitude: 33.050121).

**Figure 6:**
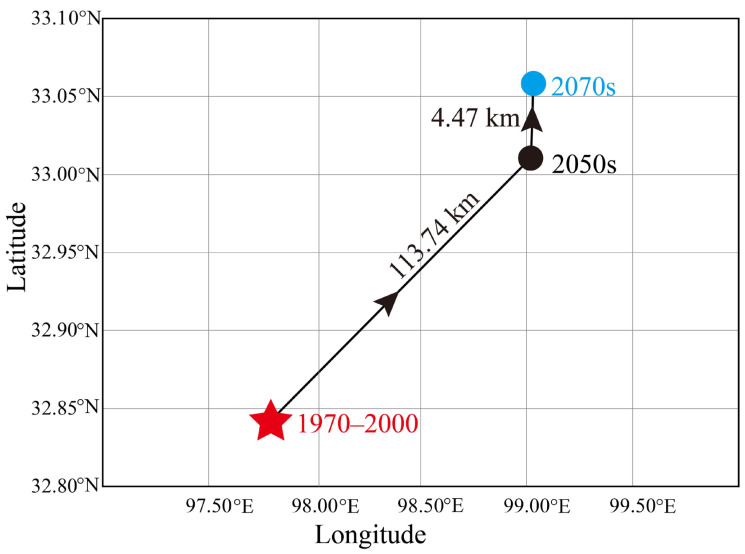
Centroid migrations of Himalayan marmots in the Qinghai–Tibet Plateau. The red star represents the centroid of the suitable habitat under the current scenarios. The black and blue circle represents the centroid of the suitable habitats in the 2050s and 2070s, respectively.

According to our study results, we found that the centroid of the suitable habitat for the Himalayan marmot moved to the northeast by 113.74 km in the 2050s, while in the 2070s, the centroid moved northeast with a migration distance of 4.47 km (Figure 6). 

## 4. Discussion

Our results suggested that the main factors affecting Himalayan marmot distribution are elevation and HFI, which are consistent with previous research that states that elevation is the main factor for Himalayan marmot distribution [29,69,70,71,72]. The Himalayan marmot has evolved a series of special physiological mechanisms to adapt to the cold, low-oxygen environment at high altitudes [73,74], which makes it unsuitable for low altitudes. However, the lack of oxygen caused at high altitudes is not suitable for the survival of the Himalayan marmot. Altitudinal factors can also affect the redistribution of water and heat conditions in local areas, with temperatures commonly decreasing at high altitudes, which, along with changes in water availability, can affect the distribution of food resources of the Himalayan marmot [75].

The HFI was a major determining factor for the geographical distribution of the Himalayan marmot, and the present study implies that this probability increased as the HFI increased. In general, wild animals prefer areas away from roads and villages to avoid human interference [76,77]. However, we found that the Himalayan marmot had the opposite preference for their habitat, which reduces the risk of predation [78,79]. In addition, the populations in the QTP areas have a concept of religion of not killing local animal species, and this may also contribute to rodents living in areas with high human activity [80]. The results of our stacking analysis suggested that the preferred habitat of the Himalayan marmot was medium-coverage grasslands, where they preferred to eat herbaceous plants [50], and that the unobstructed views in grasslands were conducive to a reduced predation risk [29,81]. 

Our model failed to quantify the impact of additional factors, such as vegetation, grazing intensity, soil features, and interspecific competition, on the potential distribution of the Himalayan marmot. Previous studies showed that vegetation was particularly crucial to habitat selection for the Himalayan marmot as vegetation cover provides hiding places that increase their chances of survival, as well as abundant food sources that increase their reproductive success [32]. In general, higher grazing intensity can reduce vegetation height, making it less suitable for the Himalayan marmot to hide [82,83]. However, lower vegetation height may provide Himalayan marmots with a greater field of view, allowing them to observe predators from a greater distance and reducing the risk of predation [84]. Soil characteristics such as type, water content, and organic matter content directly affected the selection and construction of Himalayan marmot caves and indirectly affected the richness of their food resources [85,86]. In addition, interspecific relationships (e.g., predation and competition) can also affect the potential distribution of the Himalayan marmot. Therefore, subsequent data analyses should overcome challenges in indicator quantification and data collection and include more factors that affect species distribution to improve the accuracy of species distribution simulations.

Owing to how different species adapt to temperature and precipitation factors, suitable habitats for wildlife on the QTP had different trends in response to future climate change, including expansion and reduction of suitable habitat areas [41,68,87]. According to our prediction results, suitable habitat areas for the Himalayan marmot are expected to slightly increase in the next 30–50 years. In the future, precipitation will increase in most areas of the QTP, which will provide plant growth with better conditions in this region and more abundant food resources for the Himalayan marmot, improving their survival environment [88]. Meanwhile, changes in vegetation structure and richness will affect the distribution and nutrition of food resources available to the Himalayan marmot, which will lead to changes between suitable habitats and unsuitable habitats. In addition, previous research showed that omitting non-climatic variables in future scenarios will lead to conservative projections [89], and we have encountered a similar challenge. We did not include the non-climatic variables available in our future scenario, such as human disturbance and vegetation data; our model of the future distribution of Himalayan marmots is therefore also conservative.

The data of migration trend analysis indicated that in response to climate warming, the Himalayan marmot will migrate to the northeast of QTP in the future. Numerous studies have shown that species will move to higher latitudes or altitudes in response to climate change [12,41,90], which is consistent with our findings that under global climate change conditions, the Himalayan marmot migrates to higher latitudes.

The Himalayan marmot has a positive effect on the promotion of nutrient cycling in grassland ecosystems and vegetation diversity improvement [12], and the distribution of some carnivorous animals that eat Himalayan marmots will also be affected accordingly [91]. As the main host animal of *Y. pestis* in the QTP, we expect the area of suitable habitat for Himalayan marmots to increase in the future, which means that herders, tourists, domestic animals, and other wildlife that overlap with their habitat risk plague infection and spreading it to new areas [29]. Therefore, our results can be used to adjust management strategies for Himalayan marmots by predicting where their suitable areas are likely to increase and when they will migrate to new habitats to reduce the risk of plague occurrence and ecosystem imbalance. Classifying plague hazard levels based on the appropriate habitat level of the Himalayan marmot represents a viable approach to disease control. Distinct plague prevention and control measures have been implemented across different suitable habitat levels. For example, in HAS, the Himalayan marmot population is managed by implementing more rigorous control measures such as drug poisoning and artificial killing. In MSA and LSA, more moderate control measures to maintain and stabilize the ecosystem include enhancing the protection of local predators and improving their environment quality, as well as modifying the Himalayan marmot habitats, e.g., by increasing vegetation height through reseeding and fertilization and diversifying plant species that are less preferred by Himalayan marmots to elevate their foraging costs [70]. In particular, relevant authorities should prioritize monitoring the Himalayan mar-mot population density in the border regions between HAS, MSA, and LSA to prevent further expansion of their distribution. Furthermore, certain departments should enhance their efforts in improving plague prevention and control along the migration routes of Himalayan marmots to mitigate potential outbreaks. Given the rapid development of QTP tourism and new media platforms, it is imperative to strengthen the dissemination of knowledge on plague prevention and control among tourists and local people through diverse channels, including online videos, newspapers, and display boards at tourist attractions [29]. However, the limited occurrence data of Himalayan marmots utilized in this study cannot perfectly model the real distribution of Himalayan marmots on the QTP due to the very limited research on Himalayan marmots at present. With the continuous deepening of research, if more occurrence data of Himalayan marmots can be obtained in the future, combined with methods such as random forests, artificial neural networks, and generalized additive models, more accurate and relevant information will be obtained.

## 5. Conclusions

Modeling the influence of global climate change on the spatial distribution of the Himalayan marmot in the QTP is an effective measure for predicting plague occurrence and ecological management in this region. In a contemporary climate scenario, where altitude and the HFI are the main environmental parameters that affect their potential distribution, Himalayan marmots have a greater preference for roosting in moderately covered grasslands. The area suitable for Himalayan marmots is expected to increase over the next 30–50 years, with global warming promoting the migration of Himalayan marmots to higher latitudes.

## Data Availability

The data from this study are available from the corresponding authors upon reasonable request.

## Supplementary Materials

The following supporting information can be downloaded at: https://www.mdpi.com/article/10.3390/ani13172736/s1. Table S1: *Marmota himalayana* occurrence coordinates on the Qinghai–Tibet Plateau. Table S2: Correlation of environment variables. Table S3: Environmental factors used in the present study. Table S4: The “ENMeval” package runs the optimal parameter results of the MaxEnt model. Table S5: High suitable areas for Himalayan marmots according to land-use types in the third phase (Area: km^2^). Table S6: High suitable areas for Himalayan marmots according to land-use types (two grades system) in the third phase. Table S7: Future habitat composition of Himalayan marmots in the Qinghai–Tibet Plateau under three future shared socio-economic pathways (for the years 2050 and 2070); HSA: high suitable area; MSA: medium suitable area; LSA: low suitable area; USA: unsuitable area. Table S8: Dictionary of terms and decoding of abbreviations.
